# The coronavirus outbreak: the central role of primary care in emergency preparedness and response

**DOI:** 10.3399/bjgpopen20X101041

**Published:** 2020-01-29

**Authors:** Catherine Dunlop, Amanda Howe, Donald Li, Luke N Allen

**Affiliations:** 1 Research Fellow, Institute of Metabolism and Systems Research, University of Birmingham, Birmingham, UK; 2 Professor of Primary Care and Immediate Past President of the World Organisation of Family Doctors (WONCA), Norwich Medical School, University of East Anglia, Norwich, UK; 3 President, World Organisation of Family Doctors (WONCA), Hong Kong, China; 4 GP Academic Clinical Fellow, Nuffield Department of Primary Care Health Sciences, University of Oxford, Oxford, UK

**Keywords:** Infectious illness, respiratory illness, health promotion and prevention, coronavirus, primary health care, general practice

## Background

On the last day of 2019, a cluster of cases of a pneumonia with unknown cause were reported by the Chinese authorities to the World Health Organization (WHO), believed to be connected to a seafood market in Wuhan, China. This market was closed the following day. On 7 January 2020, a novel coronavirus was isolated, and known pathogens were ruled out.^1^

Coronaviruses usually cause respiratory illness ranging from the common cold to severe acute respiratory syndrome (SARS). Clinical symptoms and signs of the Wuhan coronavirus include fever, with some sufferers experiencing difficulty breathing and bilateral pulmonary infiltrates seen on chest X-ray. WHO are referring to it as ‘2019-nCov’.

At the time of writing, there have been over 4,500 confirmed cases and 106 deaths, including among healthcare workers. Over 98% of these cases are within mainland China, but cases have also been confirmed in tens of other countries.

## Containing the outbreak

There is international concern regarding the potential spread of the virus, especially given Wuhan’s status as a major domestic and international hub, its large population of 11 million, and current Chinese New Year celebrations. To curb the spread, all public transport and air travel to and from Wuhan has been stopped, with other Chinese cities following suit. International exit screening of travellers with health information dissemination has begun, including at Heathrow airport. Contacts of confirmed cases are recommended to remain under medical observation, avoiding travel within the viral incubation period of 14 days.^2^

Whilst the mortality and infectivity of the 2019-nCoV are still unknown,^3^ there is understandable interest and concern regarding the potential for a pandemic as experienced in 2009 with H1N1 (‘swine flu’). However, the WHO decided *against* declaring a public health emergency of international concern on 22 January. Current data suggests the 2019-nCoV has a lower mortality than SARS (another coronavirus), which killed nearly 800 people globally before being contained in 2003.

Despite current reassurances about the severity of 2019-nCoV, it will be incredibly difficult to further contain the cross-border spread of the virus due to the sheer volume of global travel. It will be necessary to coordinate a unified, global response across many differing geopolitical boundaries, political settings, cultures, and health system contexts. The fact that incubating cases may have left Wuhan before the quarantine began complicates matters further, as does the emerging possibility that some carriers may be asymptomatic. WHO has asked all countries to prepare for cases including through surveillance, tracing, treatment and isolation practices, and by sharing data.^4^ The resources needed to do this effectively may not be routinely available, however, particularly in low-resource settings.

## The Chinese response

In the past decade, there has been an increased Chinese political focus on health, both nationally and internationally. Examples of a growing role in global health include unprecedented involvement in managing the Ebola outbreak and the ambitious ‘One Belt One Road’ 64-country development initiative, which encompasses 66% of the world’s population and a third of global gross domestic product.^5^

Chinese authorities were quick to close the Wuhan market, conduct epidemiological assessments, and notify the global health community.^6^ The genetic sequence of the virus was shared by Chinese authorities on the 12 January to aid international development of effective diagnostics.^1^ President Xi Jinping is personally leading a special cross-departmental taskforce, involving the heads of the health, security, traffic, education, and commerce departments.

## WHO response

The WHO exists to coordinate and lead in global health, and for the past week the Strategic Health Operations Centre (SHOC) has been holding hourly meetings to keep abreast of nCoV developments. The SHOC coordinates information and responses through a network of WHO teams, member states, and partner organisations; providing advice, tracking cases, and monitoring essential resources in the field. An Emergency Committee advises the Director General, who makes the ultimate decision on when and whether to declare a public health emergency of international concern. Thus far, escalation to this status has not been made, but the committee has planned to reconvene only days after their initial meeting to review this decision, closely following the rapid development of events.^4^

## Domestic implications

The UK government is tracing all those who have arrived from Wuhan within the past 14 days. To date, 31 completed tests on suspected cases have been returned negative and Public Health England (PHE) is advising that the risk to the public remains low. Due to international travel, as well as widespread media and public interest, the number of suspected cases is anticipated to rise and confirmed cases in the UK may yet be seen.

It is reasonable to expect that the widespread attention will impact on the demand for appointments in GP practices in coming weeks, as well as anxiety for members of the public. PHE has advised that any person coughing, feeling short of breath, or experiencing fever should seek medical attention if they have recently travelled to Wuhan. Interim guidance has been issued specifically for primary care by PHE.^7^ In summary, unwell patients with a relevant travel history should isolate themselves and consult remotely. If they come to the practice, they should be isolated on entrance to the GP surgery. If the consultation has already begun, the healthcare practitioner should leave the room, close the door, and wash their hands thoroughly with soap and water. Clinical history can be conducted by telephone, and physical examination should be avoided. If possible, arrangements should be made for any further assessment to occur in secondary care, after first discussing the differential with the hospital (and ambulance team if required). The local PHE health protection team must be informed. Generally, coronaviruses show more severe symptoms in those with chronic diseases, immunosuppression, or who are older, so these individuals may be more at risk of severe progression.

## The central role of primary care

Whilst strong epidemiology and surveillance systems are indispensable tools for the detection and monitoring of outbreaks and public health emergencies, strong primary care systems form the foundation of any emergency response. In the UK, primary care handles over 95% of all health system activity. WHO member states have repeatedly affirmed their commitment to developing their primary care systems with a view to training up community-based health professionals who are able to provide care across the spectrum of prevention, preparedness, response, and recovery. As the ‘front door’ of the health system, primary care professionals should be involved in planning and action for health emergency risk management. WONCA (the global professional body for family medicine) has actively championed the ways in which primary care can be supported to deliver care during population emergencies. National primary care bodies can coordinate with public health leads to cascade information to practitioners, communicate with the public, and collate health intelligence from the frontline primary care.^8^

The Ebola crisis taught us a valuable lesson about what happens when an outbreak takes health workers away from core functions to focus on crisis response; the number of people who died from reduced access to usual care probably exceeded the number killed by the virus.^9^ Strong health systems built on comprehensive primary care are able to integrate both functions, disseminating the emergency response resources and information required to community-level staff who have the breadth of training required to manage new suspected cases alongside routine family medicine. Decent access to primary health care is essential in health emergencies, and its infrastructure crucial for containment,^10^ just as good access to high-quality primary care is at the foundation of any strong health system.^11^
